# Safety and Effectiveness of Fast-Track Systems in Pediatric Urgent Care Units: A Systematic Review

**DOI:** 10.7759/cureus.105890

**Published:** 2026-03-26

**Authors:** Mohammad Alahmadi

**Affiliations:** 1 Pediatrics, King Abdulaziz University, Jeddah, SAU

**Keywords:** efficiency, emergency service, hospital, length of stay, organizational, patient safety, pediatrics, triage, waiting times

## Abstract

Emergency department (ED) overcrowding and prolonged waiting times remain major challenges in pediatric emergency care. This systematic review evaluated the effectiveness and safety of fast-track systems implemented in pediatric urgent care and emergency department settings. A systematic search of PubMed/MEDLINE, Embase, CINAHL, and the Cochrane Central Register of Controlled Trials was conducted for studies published between 2014 and 2024. Screening and selection followed Preferred Reporting Items for Systematic Reviews and Meta-Analyses (PRISMA) 2020 guidelines. A total of 1,038 records were identified, of which 251 were screened after duplicate removal, and 14 studies met the inclusion criteria. The included studies were conducted primarily in North America, Europe, Asia, and Australia and consisted mainly of before-and-after quality improvement (QI) studies and retrospective cohort designs.

Across the included studies, fast-track implementation was associated with improvements in operational efficiency. Reported reductions in length of stay (LOS) ranged from 8.9% to 36%, while waiting times decreased in several studies, including reductions in arrival-to-provider time from 62 to 39 minutes in redesigned triage systems. Improvements in patient flow metrics, including reduced left-without-being-seen (LWBS) rates and decreased short-stay admissions, were also observed. Safety outcomes were reported in a subset of studies and generally included 72-hour return visits or 30-day readmissions, with no statistically significant increases observed following fast-track implementation. Four studies also reported improvements in caregiver satisfaction.

Fast-track systems in pediatric emergency settings are associated with improved operational performance, particularly reductions in length of stay and waiting times, while maintaining stable safety outcomes. The effectiveness of these systems appears to depend on implementation characteristics such as dedicated clinical space, appropriate staffing models, and standardized clinical protocols. Further research is needed to evaluate long-term safety outcomes, sustainability, and applicability in diverse healthcare settings.

## Introduction and background

Emergency departments (EDs) worldwide face significant challenges related to overcrowding, prolonged waiting times, and extended lengths of stay (LOS). Globally, ED visits continue to rise, with over 130 million visits annually in the United States alone, and children accounting for approximately 20%-25% of all emergency visits [1-3]. Studies indicate that 30%-40% of emergency departments report frequent overcrowding, which contributes to delays in care and reduced operational efficiency [1,2]. These pressures are particularly pronounced in pediatric emergency settings, where seasonal surges in respiratory and infectious diseases, such as influenza and respiratory syncytial virus (RSV), significantly increase patient volumes and strain resources [3]. As a result, prolonged waiting times exceeding four hours occur in up to 25%-35% of ED visits in busy hospitals, and crowding conditions contribute to left-without-being-seen (LWBS) rates ranging from 3% to 10% depending on patient volume and hospital capacity [2,4]. Overcrowding is therefore not an operational issue but a critical patient safety concern, as it has been associated with delays in diagnosis and treatment, increased LWBS rates, and reduced patient and family satisfaction [2,4]. In pediatric populations, these operational pressures may also compromise timely triage, prolong observation periods, and delay access to specialized pediatric services.

To address these challenges, healthcare systems have implemented several operational strategies aimed at improving patient flow and reducing bottlenecks in emergency care. Among these interventions, fast-track systems have emerged as one of the most widely adopted models. In general, fast-track systems involve the creation of a parallel, expedited care pathway within or adjacent to the main emergency department to manage selected groups of patients whose clinical needs can be addressed efficiently [5]. These systems typically prioritize patients with low-acuity conditions, often identified through standardized triage tools such as the Emergency Severity Index (ESI), but may also be adapted for specific clinical populations, including children with asthma exacerbations, mental health crises, minor injuries, or other well-defined clinical conditions [6-8].

Operationally, fast-track models function by streaming eligible patients into a dedicated care pathway staffed by specialized personnel, which may include advanced practice providers, nurse practitioners, physicians, or multidisciplinary teams working under standardized clinical protocols [9,10]. By separating lower-complexity cases from the main emergency care stream, fast-track systems allow emergency departments to manage patient demand more efficiently while maintaining access for higher-acuity cases. In practice, fast-track units may involve dedicated physical spaces, rapid triage algorithms, specialized clinics for particular conditions, or protocol-driven pathways integrated within existing ED workflows.

The primary expected benefits of fast-track systems are improvements in operational performance indicators, including reductions in LOS, shorter waiting times (e.g., arrival-to-provider time), improved patient throughput, and decreased LWBS rates [11,12]. These metrics are commonly used to assess ED efficiency and patient flow. However, because fast-track systems accelerate patient assessment and treatment processes, concerns have been raised regarding potential unintended consequences for patient safety. Potential risks include inappropriate patient triage, missed diagnoses, premature discharge, or increased rates of return visits and readmissions if care processes become overly expedited [10,13]. For this reason, safety outcomes such as unplanned return visits, readmissions, adverse events, and diagnostic errors are increasingly recognized as important indicators when evaluating emergency department flow interventions.

Although previous systematic reviews have examined triage-related interventions and strategies to improve ED patient flow in general emergency care settings [1,14], evidence specific to pediatric emergency care remains comparatively limited. Pediatric EDs differ from adult EDs in several important respects, including patterns of disease presentation, caregiver involvement in care decisions, and the need for age-specific clinical expertise. Furthermore, fast-track systems implemented in pediatric settings vary substantially in design, staffing models, eligibility criteria, and clinical focus. Some systems focus on triage-based low-acuity patient streaming, whereas others involve condition-specific fast-track pathways, such as those designed for asthma management, mental health evaluation, trauma assessment, or surgical conditions. These variations make it important to synthesize available evidence to determine whether fast-track models consistently improve efficiency while maintaining patient safety in pediatric contexts.

Therefore, this systematic review aims to synthesize and critically evaluate the existing evidence regarding the effectiveness and safety of fast-track systems in pediatric urgent care and emergency department settings. Specifically, the review examines whether the implementation of fast-track pathways improves operational outcomes such as LOS, waiting times, and patient flow while maintaining acceptable safety outcomes, including rates of return visits, readmissions, and adverse events. By synthesizing findings from diverse clinical settings and fast-track models, this review seeks to provide a clearer understanding of the potential role of fast-track systems in improving pediatric emergency care delivery.

## Review

Methods

This systematic review was conducted in accordance with the Preferred Reporting Items for Systematic Reviews and Meta-Analyses (PRISMA) 2020 guidelines [15] to ensure a transparent and reproducible process.

Eligibility Criteria

Studies were selected based on the following PICOS framework [16]. Population (P)included pediatric patients (aged 0-18 years) presenting to hospital-based urgent care units, emergency departments (EDs), or dedicated pediatric emergency settings. Intervention (I) involved the implementation of any fast-track system, defined as a parallel, expedited care pathway for a select patient population within or adjacent to the main ED. This included, but was not limited to, dedicated fast-track areas, nurse-led clinics, condition-specific pathways (e.g., for asthma, mental health, and trauma), and triage-based streaming models. Comparison (C) was standard care without a fast-track system, typically represented by pre-implementation historical controls or concurrent standard care cohorts. Outcomes (O) considered were primarily effectiveness (LOS, waiting times, such as arrival-to-provider time, LWBS rates, and patient throughput) and safety (rates of missed diagnoses, adverse events, unplanned return visits, within 72 hours and 30 days, and readmissions). Patient and family satisfaction was also considered a secondary safety-related outcome. Study design (S) included original research studies, including randomized controlled trials, quasi-experimental studies, retrospective and prospective cohort studies, before-and-after quality improvement (QI) studies, and interrupted time series analyses. Reviews, commentaries, and non-English language studies were excluded.

Information Sources and Search Strategy

A systematic literature search was performed across multiple electronic bibliographic databases, including PubMed/MEDLINE, Embase, CINAHL, and the Cochrane Central Register of Controlled Trials. The search strategy utilized a combination of controlled vocabulary (e.g., MeSH terms) and keywords related to three core concepts: “fast-track,” “pediatric,” and “emergency department.” Search terms included, but were not limited to, “fast track,” “rapid assessment,” “triage,” “streaming,” “pediatric emergency service,” “emergency care,” “child,” and “adolescent.” The search was restricted by publication date, aiming to capture the most recent literature published between 2014 and 2024. The reference lists of included studies and relevant review articles were also hand-searched to identify additional eligible publications.

The following is an example of the search string used in PubMed: (“emergency department” OR “emergency room” OR “urgent care”) AND (“fast track” OR fast-track OR triage OR streaming OR “rapid assessment”) AND (waiting OR “waiting time” OR “length of stay” OR LOS OR delay OR overcrowding OR throughput OR “patient flow”) AND (child OR children OR pediatric OR paediatric OR infant OR adolescent).

Study Selection and Data Extraction

**Figure 1:**
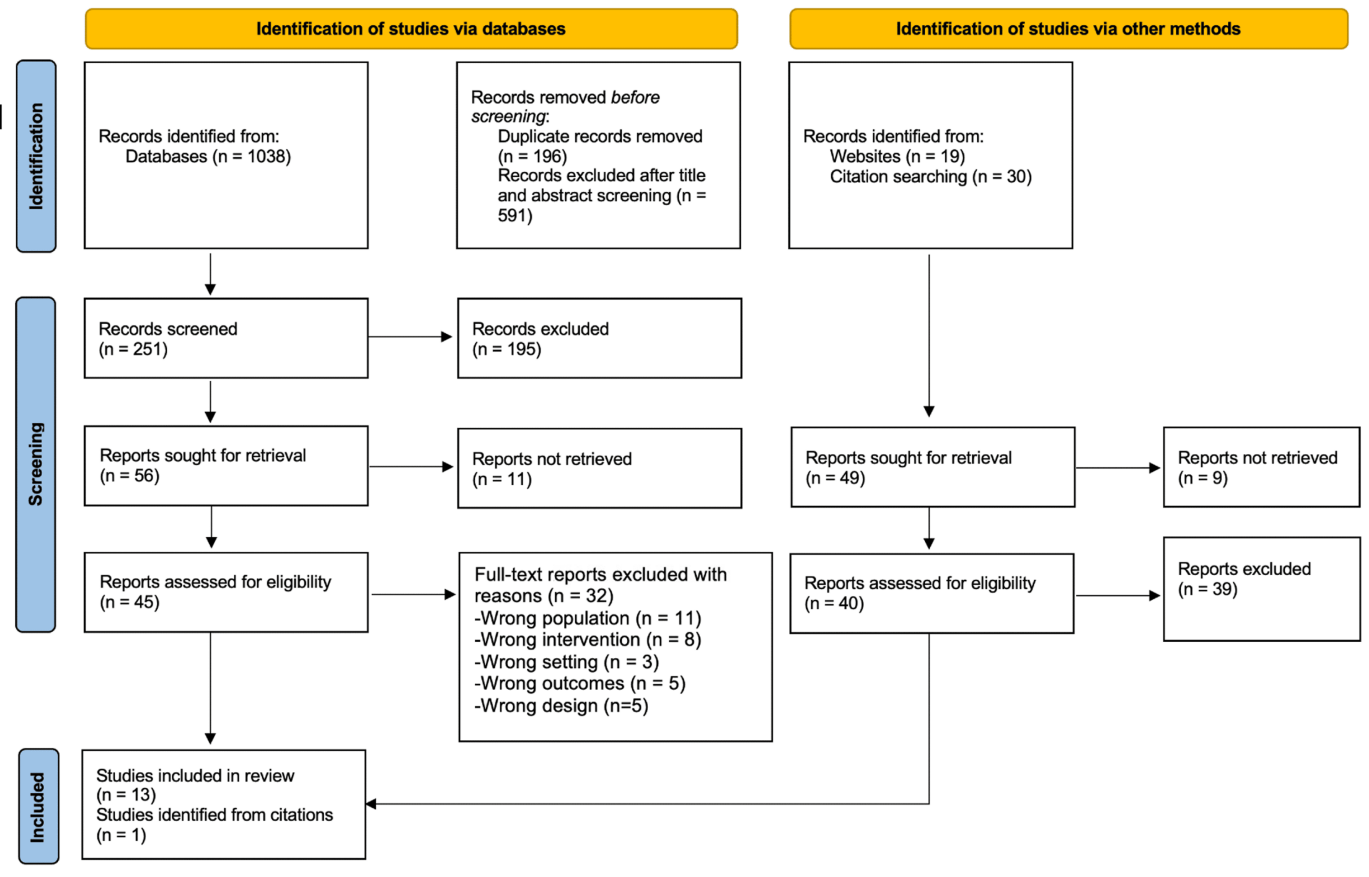
PRISMA flowchart showing the study selection process Adapted from PRISMA 2020 guidelines [15] and PRISMA flow diagram template [17] PRISMA: Preferred Reporting Items for Systematic Reviews and Meta-Analyses

The study selection process was performed by two independent reviewers who screened the titles and abstracts of all identified records against the eligibility criteria. Then, the full texts of potentially relevant studies were retrieved and assessed in detail for final inclusion. The PRISMA diagram [17] below illustrates the study selection process (Figure 1).

The extracted information included the following: (1) study characteristics (author, year, country, setting, and design), (2) population details (sample size, age, condition/focus, and eligibility criteria), (3) intervention characteristics (fast-track model, staffing, workflow, and technology used), and (4) quantitative and qualitative results for all pre-specified effectiveness and safety outcomes.

Quality Assessment (Risk of Bias)

The risk-of-bias assessment was conducted independently by two reviewers using standardized assessment tools appropriate to the study design. For before-and-after quality improvement studies, the National Institutes of Health (NIH) Quality Assessment Tool for Before-After Studies With No Control Group [18] was applied, while cohort studies were evaluated using the Newcastle-Ottawa Scale (NOS) [19]. Each study was assessed across predefined domains, including selection bias, confounding, outcome measurement, and completeness of reporting. Disagreements between reviewers were resolved through discussion to achieve consensus. When consensus could not be reached, a third reviewer independently evaluated the study and made the final determination.

Data Synthesis

A narrative synthesis was performed, and due to heterogeneity observed in intervention models, outcome definitions, study populations, and reporting metrics, the meta-analysis was not performed. The findings were structured around the key outcome themes (effectiveness and safety) and the influencing implementation characteristics (e.g., dedicated space, staffing, and protocols). Results were tabulated and summarized descriptively to identify consistent patterns, divergences, and overarching conclusions regarding the impact of fast-track systems in pediatric urgent care.

Results

The included studies span diverse pediatric emergency and urgent care contexts, predominantly from high-income countries, and employ mainly before-and-after quality improvement or retrospective cohort designs. Sample sizes vary widely, ranging from small, focused cohorts (e.g., oncology and mental health fast-tracks) to very large administrative datasets comprising over 100,000 visits. Populations include low-acuity general pediatric patients, trauma cases, respiratory and asthma presentations, oncology complications, and surgical conditions such as appendicitis. Interventions differ significantly (ranging from triage redesign and specialized care pathways to nurse-led clinics and virtual observation systems), reflecting broad heterogeneity in clinical focus, implementation models, and operational settings (Table 1).

**Table 1:** Characteristics of the included studies *Year of publication, CDU: clinical decision unit, CPETS: Chinese Pediatric Emergency Triage System, CT: computed tomography, ED: emergency department, ESI: Emergency Severity Index, FAST: Focused Assessment With Sonography for Trauma, ICD-9: International Classification of Diseases, 9th Revision, IRAS: Indice de la Respiration Aiguë Standardisé (Standardized Acute Respiratory Index), KT: knowledge transfer, MSK: musculoskeletal, PDSA: plan-do-study-act, QI: quality improvement, USA: United States of America, UK: United Kingdom

Study (author, year*)	Country/setting	Study design	Population and eligibility	Sample	Condition/focus
Martin et al. (2021) [6]	Pediatric ED (USA)	QI before-after	Low-acuity pediatric patients (ESI 4-5)	-	General low-acuity conditions
Lam et al. (2024) [20]	Pediatric ED (USA)	QI using PDSA	Patients needing ≤1 hour additional care	-	Mixed low-acuity
Manno et al. (2015) [11]	Mixed adult-pediatric ED (Italy)	Retrospective observational	Low-acuity illness/injury	3-year dataset	Mixed categories
Sheridan et al. (2016) [7]	Pediatric ED (USA)	Retrospective pre-post	Children with mental health ICD-9 codes	83 encounters in the year prior to and 129 encounters	Pediatric mental health
Nti et al. (2019) [21]	Pediatric trauma center (USA)	Retrospective chart review	Blunt abdominal trauma	CT: 413, FAST: 652	Trauma
Wong et al. (2020) [22]	Pediatric oncology fast-track clinic (Australia)	Quasi-experimental	Post-chemotherapy complications	100 prospective + 196 retrospective	Oncology complications
Cloutier et al. (2019) [5]	Pediatric ED (Canada)	Before-after KT implementation	Asthma/respiratory distress (IRAS 4-6)	134	Respiratory acute care
Lin et al. (2016) [12]	Pediatric ED (China)	Before-after implementation	All triage levels (1-5)	114,040 control, 102,969 CPETS group	General pediatric
McCoy et al. (2018) [8]	Children’s hospital ED (USA)	QI (PDSA cycles)	Asthma exacerbations	-	Asthma
Birahinduka and Stewart (2014) [23]	Pediatric ED (UK)	Before-after	Low-acuity MSK injuries	200 visits	Musculoskeletal injuries
Berkowitz et al. (2018) [24]	Pediatric level 1 trauma ED (USA)	QI initiative	ESI level 4-5	-	Low-acuity flow redesign
Lam et al. (2021) [25]	Pediatric surgery/ED (USA)	Retrospective pre-post	Simple/complicated appendicitis	276	Appendicitis
Karacabeyli et al. (2019) [26]	Pediatric ED CDU (Canada)	Retrospective cohort	Patients eligible for <24-hour observation	1,696	Mixed diagnoses
Cator et al. (2014) [27]	Pediatric ED (USA)	Retrospective administrative analysis	9 common pediatric diagnoses	1,614 visits in year 0 and 1,510 visits in year 1	Virtual observation

Across 14 included studies published between 2014 and 2024, evidence demonstrates substantial heterogeneity in fast-track design, operational processes, eligibility criteria, and evaluation methodology.

Effectiveness of Fast-Track Systems

Reductions in LOS: Of the 14 included studies, nine reported reductions in LOS, and three reported minimal or no significant change. For example, Martin et al. [6] reported a 36% reduction in LOS among low-acuity pediatric patients (144 to 92 minutes), while Lam et al. [20] demonstrated a reduction from 121 to 103 minutes following the introduction of a Supertrack process. Sheridan et al. [7] also observed a 27% decrease in LOS for pediatric mental health presentations. In contrast, Birahinduka and Stewart [23] reported an increase in LOS from 50.8 to 69.4 minutes, which the authors attributed to inefficiencies introduced by a dual-tracking system. Across studies that reported inferential statistics, several demonstrated statistically significant improvements (p < 0.05) in LOS or waiting times, including those by Manno et al. [11], Sheridan et al. [7], and Lin et al. [12]. However, some quality improvement studies reported improvements descriptively without formal statistical testing, reflecting the operational nature of many fast-track evaluations.

Improvements in waiting times: Four studies demonstrated statistically significant reductions in waiting times (p < 0.05), including those by Lin et al. [12], Berkowitz et al. [24], Manno et al. [11], and Nti et al. [21]. These studies reported improvements such as reduced arrival-to-provider times, faster access to diagnostic imaging, and shorter triage-to-treatment intervals. In addition to these statistically significant findings, several other studies described improvements in waiting times descriptively without providing inferential statistical testing, which is common in quality improvement-based evaluations.

Impact on patient flow and system efficiency: Five studies [11,21,12,25,26] reported statistically significant improvements in system efficiency metrics, including reductions in leave-without-being-seen (LWBS) rates, improved patient throughput, decreased admission rates, or reductions in short-stay hospitalizations (p < 0.05). These improvements were observed in studies evaluating multi-specialty fast-track models, streamlined triage systems, and clinical decision units adjacent to pediatric emergency departments [11,12,26]. Two additional studies [8,24] reported improvements in patient flow metrics descriptively without formal statistical testing, indicating trends toward enhanced operational performance following fast-track implementation. One study [23] reported no meaningful improvement in system efficiency outcomes, suggesting that the impact of fast-track models may depend heavily on implementation design, staffing structure, and integration with existing emergency department workflows. Table 2 shows further details of the effectiveness of fast-track systems.

**Table 2:** Effectiveness of fast-track systems across studies *Year of publication, CDU: clinical decision unit, CT: computed tomography, ED: emergency department, FAST: Focused Assessment With Sonography for Trauma, LOS: length of stay, LWBS: left without being seen

Study (author, year*)	LOS	Waiting time	Patient flow/system efficiency
Martin et al. (2021) [6]	Reduced from 144 to 92 minutes (36% reduction)	-	Improved throughput for low-acuity patients
Lam et al. (2024) [20]	Reduced from 121 to 103 minutes	-	Increased proportion of timely discharges
Manno et al. (2015) [11]	LOS reduction reported (raw values not provided)	Waiting time reduced (p < 0.01)	LWBS rates significantly reduced (p < 0.01)
Sheridan et al. (2016) [7]	LOS reduced by 27% (p < 0.01)	-	Psychiatric hospitalization rates decreased
Nti et al. (2019) [21]	-	CT acquisition time reduced from 37 to 28 minutes (p < 0.05); FAST reduced from 18 to 8 minutes (p < 0.05)	Improved trauma imaging workflow
Wong et al. (2020) [22]	-	-	Reduced laboratory interventions compared with ED care (p = 0.0027)
Cloutier et al. (2019) [5]	LOS decreased by 23 minutes	-	LWBS rates decreased (not statistically reported)
Lin et al. (2016) [12]	Not reported	Waiting time reduced from 41.6 to 37.3 minutes (p < 0.05)	Improved triage accuracy and patient flow
McCoy et al. (2018) [8]	No significant change in LOS	Respiratory clinical score assessment < 20 minutes; steroid administration < 60 minutes	Asthma admissions reduced from 24% to 17%
Birahinduka and Stewart (2014) [23]	Increased from 50.8 to 69.4 minutes (p = 0.01)	Imaging waiting time increased from 30.9 to 58.5 minutes	Patient flow worsened due to dual-track workflow
Berkowitz et al. (2018) [24]	LOS reduced by 11 minutes	Arrival-to-provider time reduced from 62 to 39 minutes	Improved flow for low-acuity patients
Lam et al. (2021) [25]	LOS reduced from 17.0 to 15.5 hours (p = 0.02)	-	Early discharges increased from 21% to 37.5%
Karacabeyli et al. (2019) [26]	Median CDU LOS of 4.4 hours	-	Short-stay hospitalizations reduced from 3.62% to 3.23% (p = 0.001)
Cator et al. (2014) [27]	ED discharge LOS reduced from 5.6 to 5.1 hours (p < 0.001)	-	Admission efficiency unchanged

Safety Outcomes

Return visits and readmissions: Three studies evaluated these safety outcomes following fast-track implementation. Among these, two studies reported no statistically significant change in return visit or readmission rates compared with pre-implementation periods [7,25]. For example, Lam et al. [25] found no significant difference in 30-day readmissions between pre- and post-fast-track appendicitis cohorts, while Sheridan et al. [7] similarly observed no increase in return emergency department visits following the implementation of a psychiatric fast-track team. In addition, one study reported a clinically related revisit rate of 9.2%, which was consistent with typical emergency department benchmarks but did not include statistical comparisons [26].

Left-without-being-seen (LWBS) rates: Among the included studies, two reported statistically significant reductions in LWBS rates following fast-track implementation [11,12]. For example, Manno et al. [11] observed significant reductions in LWBS rates after implementing a multi-specialty fast-track model within a mixed adult-pediatric emergency department. Similarly, Lin et al. [12] reported improved triage efficiency and reductions in patient flow congestion, which were associated with lower LWBS rates following implementation of the Chinese Pediatric Emergency Triage System (CPETS). In addition, one study reported reductions in LWBS descriptively without reporting statistical testing [5].

Patient and family satisfaction: Improvements in patient and family satisfaction were reported in four of the reviewed studies. Lin et al. [12] found higher caregiver satisfaction under a CPETS, while Wong et al. [22] reported increased satisfaction among parents and patients with a nurse-led oncology fast-track model. Cloutier et al. [5] noted high acceptability among families, and McCoy et al. [8] provided indirect indications of increased satisfaction through the reduction of treatment delays.

While other studies reported stable safety outcomes following fast-track implementation, one study reported negative operational consequences. Birahinduka and Stewart [23] observed that the introduction of a general practitioner (GP)-led urgent care center (UCC) for pediatric musculoskeletal injuries resulted in increased length of stay (50.8 to 69.4 minutes) and longer imaging waiting times (30.9 to 58.5 minutes), which the authors attributed to workflow fragmentation created by a dual-tracking system

Implementation Characteristics Affecting Outcomes

Dedicated space versus integrated fast-track models: The physical and operational design of the fast-track system had a discernible impact on its success. Outcomes were generally better when systems included a dedicated treatment space, clearly designated staff, and explicit patient eligibility criteria. These elements created a self-contained and efficient care environment. Systems that operated without a dedicated area, such as the model described by Lam et al. [20], still demonstrated benefits but typically yielded smaller operational gains compared to their dedicated counterparts.

Staffing models: The composition and adequacy of the clinical team were directly linked to the efficiency and success of fast-track models. Successful implementations frequently involved the use of advanced practice providers, specialized nurses, and dedicated mental health professionals. Furthermore, multidisciplinary teams tailored for specific conditions such as respiratory illnesses or trauma proved highly effective. On the other hand, inadequate or fragmented staffing was a limiting factor; for example, the GP-led urgent care center (UCC) model was correlated with worsened performance metrics [23], underscoring the importance of appropriate and integrated staffing.

Protocols, algorithms, and clinical pathways: The use of structured protocols, algorithms, and clinical pathways was a cornerstone of successful fast-track systems. In areas such as pediatric asthma, appendicitis, and trauma, these standardized tools facilitated greater consistency in care, reduced time to critical treatments, and improved overall care coordination. In the case of appendicitis, structured pathways also contributed to improved antimicrobial stewardship. The presence of clear guidelines ensured that patients received evidence-based care efficiently, regardless of the individual clinician.

Use of technology: Technology played a pivotal role in enhancing the functionality of fast-track systems. Interventions that utilized technology, such as specialized CPETS software, electronic reminders, structured electronic medical record (EMR) order sets, and digital education tools, demonstrated improved triage accuracy, better adherence to clinical guidelines, and smoother patient flow. By embedding decision support into the workflow, technology helped to standardize processes and reduce variability, thereby supporting both safety and efficiency goals (Table 3).

**Table 3:** Implementation characteristics affecting outcomes *Year of publication, ACS: American College of Surgeons, CPETS: Chinese Pediatric Emergency Triage System, CDU: clinical decision unit, ED: emergency department, EHR: electronic health record, EMR: electronic medical record, ESI: Emergency Severity Index, FAST: Focused Assessment With Sonography for Trauma, GP: general practitioner, ICD-9: International Classification of Diseases, 9th Revision, IRAS: Indice de la Respiration Aiguë Standardisé (Standardized Acute Respiratory Index), MD: medical doctor, MSK: musculoskeletal, NP: nurse practitioner, OB-GYN: obstetrics and gynecology, OR: operating room, PEM: pediatric emergency medicine, PEDS: pediatrics, RN: registered nurse, RCS: respiratory clinical score, REDCap: Research Electronic Data Capture, RT: respiratory therapist, SPC: statistical process control

Study (author, year*)	Fast-track model	Staffing structure	Eligibility criteria	Workflow processes	Technology/tools used
Martin et al. (2021) [6]	Dedicated fast-track area	Advanced practice provider + RN	ESI 4-5 low-acuity	Evaluation in a dedicated zone	-
Lam et al. (2024) [20]	Supertrack without dedicated space	Existing ED staff	Patients needing ≤1 hour of care	Rapid triage then primary ED management	Reminders, intake huddles, prompts
Manno et al. (2015) [11]	Specialized mini-clinics (peds, OB-GYN, trauma, primary care)	Existing specialists	Low-acuity presentations	Streaming to specialty care pods	-
Sheridan et al. (2016) [7]	Dedicated psychiatric fast-track	Psychiatrist + social worker	Pediatric mental health ICD-9 codes	Mental health evaluation	None
Nti et al. (2019) [21]	Streamlined trauma response	ED physicians, pediatric surgeons, residents	Trauma requiring activation	Timed imaging and OR access	FAST equipment, ACS guidelines
Wong et al. (2020) [22]	Nurse-led oncology fast-track	Oncology nurses	Post-chemotherapy symptoms	Rapid review and reduced interventions	-
Cloutier et al. (2019) [5]	Respiratory fast-track zone	Nurses, physicians, respiratory therapists	Moderate IRAS score (4-6)	Nurse-led evaluation, interdisciplinary support	Digital tablets, educational tools
Lin et al. (2016) [12]	CPETS structured triage system	Triage nurses	Triage levels 1-5	Software-guided triage and monitoring	CPETS triage software
McCoy et al. (2018) [8]	Standardized asthma pathway	Nurses, RTs	Asthma exacerbations	RCS scoring tool, early treatment	EMR order sets
Birahinduka and Stewart (2014) [23]	GP-led urgent care center	General practitioners	Low-acuity MSK injuries	Dual-tracking workflow	Adastra electronic records system
Berkowitz et al. (2018) [24]	Front-end redesign with fast-track rooms	Additional MD/RN	ESI 4-5	Pivot-nurse model, rapid triage	EHR + SPC analytics
Lam et al. (2021) [25]	Surgical fast-track pathway	Nurses using standardized orders	Simple appendicitis	Nurse-led discharge protocols	EMR
Karacabeyli et al. (2019) [26]	CDU (4-bed)	RN + NP or PEM physician	Expected discharge <24 hours	Standardized CDU order sets	REDCap
Cator et al. (2014) [27]	Virtual observation	ED clinicians	9 common pediatric diagnoses	Standardized order sets	-

Variations Across Clinical Conditions

While the majority of fast-track systems are designed for high-volume, low-acuity presentations, the evidence demonstrates significant adaptability to specialized patient populations. Several studies successfully implemented tailored models for specific clinical conditions, yielding positive outcomes. In mental health, dedicated psychiatric fast-track teams were effective in improving length of stay and hospitalization rates. For trauma patients, streamlined fast-track processes significantly reduced delays in obtaining essential imaging. In the management of pediatric asthma and respiratory distress, the use of standardized clinical pathways within a fast-track framework consistently improved time-to-treatment and reduced admission rates. Similarly, in oncology, nurse-led fast-track clinics proved beneficial by reducing unnecessary investigations and enhancing patient satisfaction. This diversity in application underscores that the fast-track principle is a versatile one, capable of being effectively tailored to both general emergency department workflows and highly specific, condition-oriented care processes.

Quality of the Evidence

**Figure 2:**
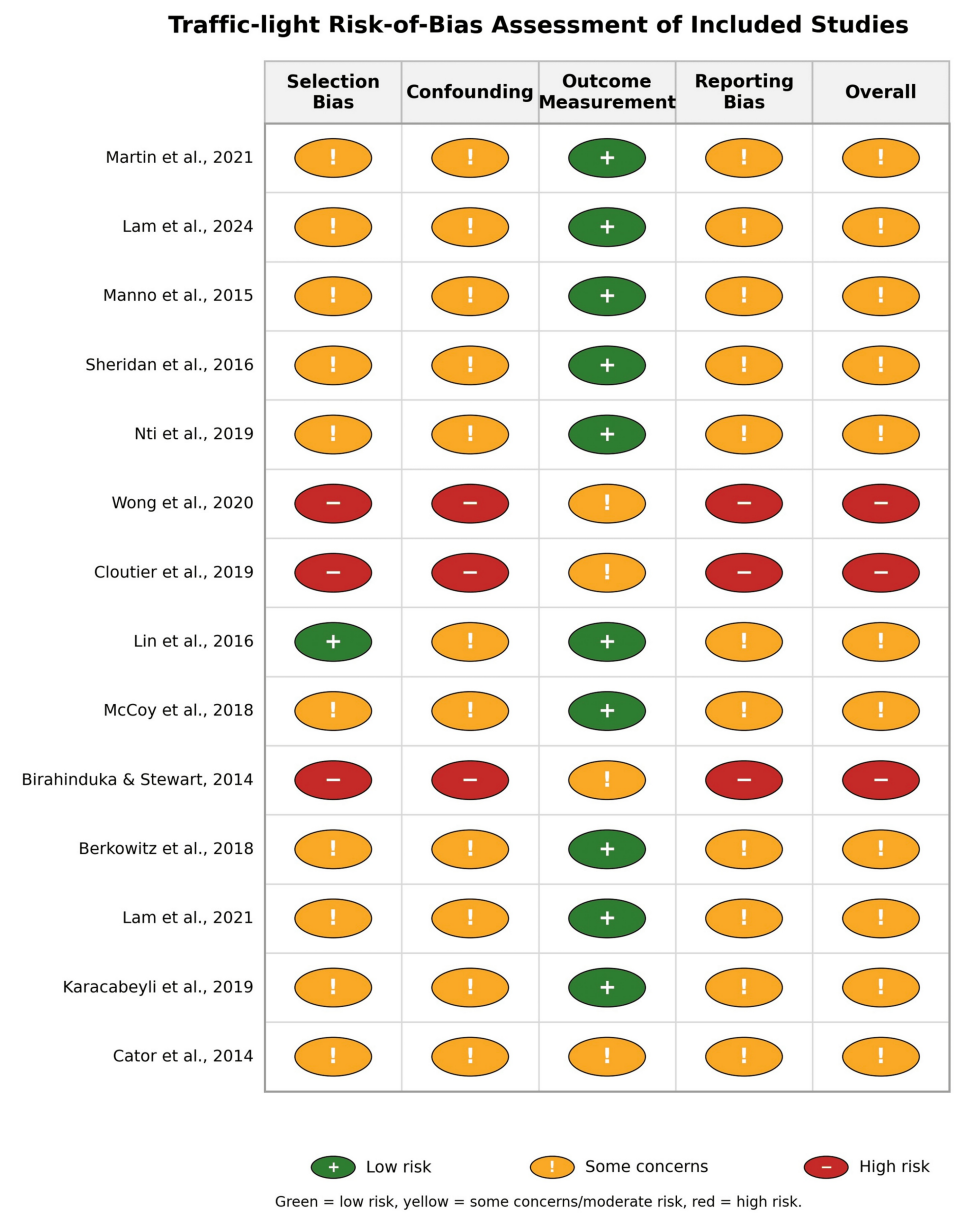
Risk-of-bias assessment of included studies Risk of bias was evaluated using the NIH Quality Assessment Tool for Before-After studies [18] and the Newcastle-Ottawa Scale for cohort studies [19]. Green indicates low risk of bias, yellow indicates some concerns (moderate risk), and red indicates high risk of bias across methodological domains.

Most studies suffered from the absence of randomization and concurrent control groups, susceptibility to time trends and confounding (policy changes, staffing, and seasonal variation), limited reporting of missing data, blinding, and adverse event surveillance. However, large database or cohort-based studies [12,26,27] had stronger selection and outcome measurement domains and are best classified as moderate risk. Small single-site QI projects and short before-and-after studies [5,22,23] are generally considered high risk, primarily due to confounding, small sample sizes, and limited reporting (Table 4, Figure 2).

**Table 4:** Quality/risk of bias assessment of the included studies Risk of bias and quality of methodology were assessed according to NIH Study Quality Assessment Tools [18] and NOS methodology [19]. *Year of publication, ACS: American College of Surgeons, CPETS: Chinese Pediatric Emergency Triage System, CDU: clinical decision unit, ED: emergency department, EHR: electronic health record, EMR: electronic medical record, ESI: Emergency Severity Index, FT: fast track, ISS: Injury Severity Score, LOS: length of stay, LWBS: left without being seen, MSK: musculoskeletal, NIH: National Institutes of Health, NOS: Newcastle-Ottawa Scale, OR: operating room, PDSA: plan-do-study-act, QI: quality improvement, RCS: respiratory clinical score, REDCap: Research Electronic Data Capture, SPC: statistical process control, UCC: urgent care center

Study (author, year*)	Design	Assessment tool used	Selection/confounding bias	Outcome measurement and data quality	Other biases (e.g., reporting, fidelity)	Overall risk of bias
Martin et al. (2021) [6]	Single-site QI before-after (low-acuity fast track)	NIH Before-After (Pre-Post) [18]	No concurrent control; possible changes in staffing/case-mix over time; convenience population of ESI 4-5	LOS was objectively measured from ED system; safety outcomes not reported; limited information on missing data	Publication focuses on positive efficiency outcomes; selective reporting of safety possible	Moderate-high
Lam et al. (2024) [20]	QI study using PDSA cycles, pre-post	NIH Before-After + QI SPC concepts [18]	Clear objective criteria but still non-randomized; potential secular trends; no external control	LOS and discharge metrics objectively recorded; no detailed safety surveillance; unclear handling of missing data	Possible reporting bias toward successful PDSA cycles; limited detail on unsuccessful cycles	Moderate
Manno et al. (2015) [11]	Retrospective observational (mixed adult-pediatric, specialized fast track)	NOS [19]	Large 3-year dataset; allocation to fast track determined by triage; no adjustment for confounders (age, acuity, time of day)	Objective time outcomes; LWBS recorded; limited detail on accuracy/completeness of data	Possible residual confounding; unclear whether other system changes occurred during study period	Moderate
Sheridan et al. (2016) [7]	Retrospective pre-post (psychiatric team)	NIH Before-After [18]	Historical control; possible changes in referral patterns, mental health services, or staffing; modest sample size	LOS and admission rate objectively recorded; no systematic adverse event/missed diagnosis monitoring	Limited detail on protocol fidelity; risk of selective emphasis on positive endpoints	Moderate-high
Nti et al. (2019) [21]	Retrospective chart review (trauma response training)	NOS [19]	Trauma team activation criteria stable, but allocation not randomized; potential co-interventions (equipment, protocols)	Imaging times, OR times are objective; ISS and severity documented; missing data risk in chart review not fully addressed	Level III evidence; short follow-up; no explicit assessment of clinical outcomes or complications	Moderate
Wong et al. (2020) [22]	Quasi-experimental (prospective clinic versus retrospective ED group)	NIH Before-After + NOS [18,19]	Non-concurrent control, small sample; oncology patients possibly systematically different between periods; selection of first 100 FT cases	Laboratory/intervention counts objectively recorded; LOS and admissions reported, but not always with full statistical detail; safety outcomes under-reported	Strong probability of selection and performance bias; satisfaction self-reported without blinding	High
Cloutier et al. (2019) [5]	Short before-after project (1 month, respiratory fast track)	NIH Before-After [18]	Very short evaluation period; high vulnerability to seasonal variation, staffing changes; moderate sample only	LOS reductions reported; LWBS reductions mentioned but with limited quantitative detail; no systematic adverse event tracking	Knowledge transfer project context may favor positive results; limited methodological reporting	High
Lin et al. (2016) [12]	Large before-after with historical comparison (CPETS triage)	NOS [19]	Very large samples; groups drawn from sequential years (control versus CPETS); remaining risk of secular trends and case-mix differences	Triage rates, accuracy, and wait times objectively recorded using software; satisfaction survey may be subject to response bias	No randomization; no risk-adjusted analysis; but robust process measures	Moderate
McCoy et al. (2018) [8]	QI pathway implementation (asthma)	NIH Before-After [18]	No concurrent control; potential changes in asthma epidemiology or ED processes; no formal adjustment for confounders	Time to RCS and steroids objective; admissions measured; readmissions tracked but limited adverse event detail	Single center; selective reporting of positive process metrics cannot be excluded	Moderate-high
Birahinduka and Stewart (2014) [23]	Small before-after evaluation (MSK injuries, UCC)	NIH Before-After [18]	200 attendances only; non-randomized; possible case-mix differences across months; minimal description of sampling strategy	Imaging and LOS times from electronic system; no description of data validation, missing data, or secondary outcomes	Limited reporting; high risk of publication and reporting bias	High
Berkowitz et al. (2018) [24]	QI system redesign (front-end flow, low-acuity)	NIH Before-After [18]	Non-controlled single-site QI; however, uses SPC methods and large volumes of ESI 4-5	Arrival-to-provider time and LOS objectively captured from EHR; 72-hour return visits tracked, but no detailed adverse event review	Lean and Kaizen processes described, but fidelity to interventions and concurrent initiatives not fully described	Moderate
Lam et al. (2021) [25]	Retrospective pre-post cohort (fast-track discharge)	NOS [19]	Clear inclusion/exclusion, defined simple versus complicated appendicitis; non-randomized historical cohorts; some risk of residual confounding	LOS, readmissions, complications extracted from EMR; clear definitions; follow-up adequate for 30-day outcomes	Single center; potential unmeasured changes in peri-operative care	Moderate
Karacabeyli et al. (2019) [26]	Retrospective cohort (CDU)	NOS [19]	Large sample of CDU patients; well-defined eligibility; however, CDU versus non-CDU allocation not randomized	Outcomes (short-stay hospitalization, LOS, revisit) well-defined; use of REDCap suggests structured data management	Single center; limited reporting on potential co-interventions or service changes	Moderate
Cator et al. (2014) [27]	Retrospective secondary analysis (virtual observation)	NOS [19]	All observation-eligible visits included; intervention group based on clinician uptake; possible selection bias and time trends	LOS and admissions derived from administrative data; coding reliability not fully reported	No detailed description of safety endpoints; some risk of residual confounding	Moderate

Discussion

This systematic review examined the effectiveness and safety of fast-track systems implemented in pediatric urgent care and emergency settings across 14 studies published between 2014 and 2024, drawing from diverse clinical contexts including low-acuity general presentations, mental health crises, trauma, oncology complications, and condition-specific pathways such as asthma and appendicitis. Across the included studies, reductions in length of stay ranged from 8.9% to 36%, with absolute reductions ranging from 23 minutes to more than 50 minutes in low-acuity patient groups. Several studies also reported meaningful reductions in waiting times, including decreases in arrival-to-provider time from 62 to 39 minutes and overall waiting time reductions from 41.6 to 37.3 minutes. These findings align with broader international evidence showing that fast-track systems are a cornerstone of modern ED flow strategies, especially for high-volume pediatric populations. For example, a systematic review by Oredsson et al. [1] reported that fast-track units substantially reduce ED LOS for non-urgent patients by pooling multidisciplinary expertise and enabling parallel processing of care. Similarly, Rowe et al. [14] revealed, in their systematic review, that pediatric-focused triage and flow redesigns significantly improved throughput without increasing adverse events, underscoring the applicability of such systems to pediatric contexts.

The current review found notable reductions in LOS across most included studies. For instance, Martin et al. [6] observed a 36% decrease in LOS for low-acuity patients following the introduction of a dedicated advanced practice provider fast track, while Lam et al. [20] demonstrated significant improvements using a Supertrack model even without a dedicated physical space. These results align with the broader literature, which shows that task-shifting to trained advanced practice providers expedites care without compromising accuracy. For example, studies in the review reported reductions in low-acuity LOS of up to 52 minutes, reductions in arrival-to-provider times exceeding 35%, and improved discharge efficiency with early discharge rates increasing from 21% to 37.5% in surgical fast-track pathways. Similarly, studies reported that nurse practitioner-led models in EDs reduced LOS and improved patient satisfaction [9,28]. Furthermore, Sheridan et al. [7] documented a 27% reduction in LOS for pediatric psychiatric visits using a dedicated mental health team, consistent with findings of previous studies that specialized mental health triage pathways reduced boarding times and admission rates [29,30]. More studies reported that nurse practitioner-led and advanced practice provider models in emergency departments reduce length of stay, maintain diagnostic accuracy, and improve patient satisfaction when supported by structured protocols and supervision [9,28,31-34]. Across four studies reporting satisfaction outcomes, caregivers consistently reported improved perceptions of timeliness, communication, and overall care experience following fast-track implementation. Collectively, these observations support the principle that condition-specific fast-track structures (whether pathway redesign, specialized teams, or triage reconfiguration) yield huge gains in efficiency when the personnel skill mix is appropriately aligned with the patient population.

Waiting times were also consistently reduced following fast-track implementation, particularly when systems integrated rapid triage algorithms or technological decision support tools. Reported reductions included decreases in overall waiting time from 41.6 to 37.3 minutes, reductions in arrival-to-provider time from 62 to 39 minutes, and reductions in imaging delays for trauma patients from 37 to 28 minutes. The CPETS model described by Lin et al. [12] demonstrated significant decreases in waiting time, including reductions for critically ill children, suggesting that fast-track triage tools can improve prioritization accuracy as well. This finding aligns with evidence that electronic triage support systems improve both speed and accuracy of pediatric triage in high-volume urban EDs [31]. Similarly, Nti et al. [21]’s evidence that trauma fast-track teams reduced imaging and operating room delays aligns with global trauma literature showing that early coordinated assessment reduces morbidity and mortality in pediatric trauma cases [32].

System efficiency metrics, including throughput and LWBS rates, also improved in most studies. Although exact percentages were not consistently reported, the available evidence indicates meaningful reductions in LWBS rates following fast-track implementation, with statistically significant improvements reported in at least two studies evaluating multi-specialty fast-track models. Reductions in LWBS, as observed by Manno et al. [11] and Cloutier et al. [5], are clinically significant because LWBS is associated with increased risk of deterioration, poor patient experience, and delayed diagnoses. Previous studies also underline this association: Pham et al. [2] found that ED crowding and long waits are strong predictors of LWBS, and the introduction of fast-track areas decreases LWBS by improving queue management. In pediatric settings specifically, earlier studies found that fast-track implementation reduced LWBS in high-volume hospitals [33,34]. Thus, the observed improvements in LWBS in the current review reflect not only efficiency gains but also enhanced access and equity in pediatric emergency care delivery. However, improved operational efficiency should not automatically be interpreted as improved equity in access to emergency care. Differences in socioeconomic status, geographic location, and health literacy may still influence which patient populations benefit most from fast-track systems. Future evaluations should therefore include equity-sensitive indicators to ensure that efficiency gains translate into equitable care delivery.

Importantly, the reviewed studies reported no increase in adverse events, return visits, or missed diagnoses. For example, Lam et al. [20] found no increase in return ED visits after Supertrack implementation, and Sheridan et al. [7] similarly observed no change in return rates after restructuring mental healthcare pathways. This aligns with other studies, which concluded that fast-track models do not increase diagnostic error rates when eligibility criteria are properly followed [10,13,35]. Moreover, evidence shows that nurse-led fast-track units maintained diagnostic safety in pediatric cohorts [36,37], indicating that with adequate clinical governance, the risk of missed diagnoses is low. However, exceptions in the reviewed studies highlight implementation challenges. The GP-led urgent care model described by Birahinduka and Stewart [23] led to increased LOS and imaging delays. Similar challenges have been documented in other contexts, where poorly integrated fast-track systems exacerbate rather than relieve bottlenecks. Previous studies found that introducing parallel care streams without sufficient staffing or clear triage protocols worsened ED flow [4,38-40], underscoring that fast-track systems are sensitive to design quality, staffing adequacy, and information flow. The negative findings by Birahinduka and Stewart [23] emphasize that physical separation or new pathways alone are insufficient without system-wide alignment and proper coordination.

Variability across clinical conditions also emerged as a central theme. Fast-track systems were most effective when tailored to specific clinical populations (respiratory distress, asthma, appendicitis, mental health, oncology, and trauma all benefited from bespoke, protocol-driven models). This observation is consistent with the literature on condition-specific ED pathways. For example, a previous review concluded that asthma pathways significantly reduce wait times and admission rates in pediatric emergency departments [41]. Likewise, standardized appendicitis care has been shown to reduce hospitalization durations and improve the value of care [42]. The consistency of these findings across both the current review and broader evidence supports the argument that condition-tailored fast tracks offer superior performance compared with generic fast-track models in specialized pediatric populations.

The role of technology emerged as a facilitating factor in several studies. Systems incorporating electronic triage, computerized decision support tools, digital educational platforms, or EMR-based order sets demonstrated superior improvements in triage accuracy, flow, and guideline adherence. These observations are in line with systematic reviews indicating that digital tools enhance triage reliability, particularly in pediatric settings where symptom presentations are heterogeneous [43-46]. Moreover, in low-resource environments, even simple digital enhancements can optimize fast-track performance. A study in Kenya by Wachira et al. [47] found that digital triage systems significantly improved throughput in pediatric EDs despite limited physical infrastructure.

For successful implementation, fast-track systems should incorporate strong quality assurance mechanisms, including clearly defined eligibility criteria, standardized triage protocols, continuous monitoring of safety indicators (such as adverse events, diagnostic errors, and return visits), and periodic workflow audits. Integrating electronic decision support tools and structured clinical pathways can further enhance both safety and efficiency.

Despite generally positive findings, some limitations should be acknowledged. Most included studies used before-after or quality improvement designs, which limit causal inference and are susceptible to confounding and temporal effects. Reporting of statistical effect measures was inconsistent, and safety outcomes, such as adverse events, diagnostic errors, or long-term patient outcomes, were rarely assessed systematically. Considerable heterogeneity was also observed across studies in terms of fast-track definitions, staffing models, clinical pathways, and outcome reporting, which limited cross-study comparability and precluded meta-analysis. Sample sizes varied substantially across studies, reflecting differences in methodological rigor. In addition, few studies evaluated the long-term sustainability of fast-track interventions. Future research should prioritize more rigorous designs, including randomized or controlled studies, standardized reporting of emergency department performance metrics, and comprehensive assessment of patient safety outcomes.

## Conclusions

Overall, the findings of this systematic review contribute to the growing body of evidence supporting fast-track systems as an effective strategy for improving pediatric urgent care efficiency without compromising safety. Across the 14 studies included in this review, fast-track implementation was associated with meaningful improvements in operational performance. Reported reductions in length of stay ranged from approximately 8.9% to 36%, corresponding to absolute reductions of 23 to more than 50 minutes in low-acuity patient groups, while waiting times decreased by up to 34% in some settings. Additional improvements were observed in system flow metrics, including reductions in left-without-being-seen (LWBS) rates and hospital admissions, as well as increased early discharge rates in condition-specific pathways. Importantly, these efficiency gains were achieved without increases in return visits, readmissions, or adverse events, suggesting that well-designed fast-track systems can enhance emergency department throughput while maintaining patient safety. Consistent with international literature, these systems offer a practical strategy for reducing overcrowding and improving patient experience in pediatric emergency care. Future research should prioritize rigorous evaluation of safety outcomes, sustainability, cost-effectiveness, caregiver experience, and the integration of digital innovations, particularly in resource-limited settings where the potential impact may be greatest.

## Figures and Tables

**Figure 3:**
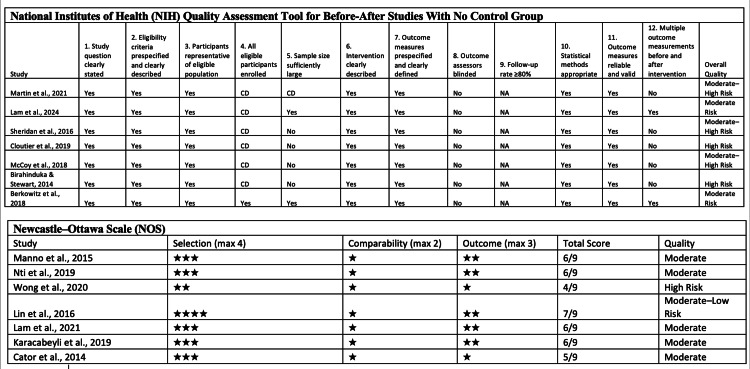
Risk of bias chart

## Appendices

Figure 3 presents the risk of bias chart using the NIH Quality Assessment Tool for Before-After Studies and the Newcastle-Ottawa Scale.
